# Neurological Complications of Middle East Respiratory Syndrome Coronavirus: A Report of Two Cases and Review of the Literature

**DOI:** 10.1155/2016/3502683

**Published:** 2016-04-28

**Authors:** Hussein Algahtani, Ahmad Subahi, Bader Shirah

**Affiliations:** ^1^King Abdulaziz Medical City/King Saud bin Abdulaziz University for Health Sciences, P.O. Box 12723, Jeddah 21483, Saudi Arabia; ^2^King Saud bin Abdulaziz University for Health Sciences, P.O. Box 12723, Jeddah 21483, Saudi Arabia; ^3^King Abdullah International Medical Research Center/King Saud bin Abdulaziz University for Health Sciences, P.O. Box 12723, Jeddah 21483, Saudi Arabia

## Abstract

Middle East Respiratory Syndrome Coronavirus (MERS-CoV) was first discovered in September 2012 in Saudi Arabia. Since then, it caused more than 1600 laboratory-confirmed cases and more than 580 deaths among them. The clinical course of the disease ranges from asymptomatic infection to severe lower respiratory tract illness with multiorgan involvement and death. The disease can cause pulmonary, renal, hematological, and gastrointestinal complications. In this paper, we report neurological complications of MERS-CoV in two adult patients, and we hypothesize the pathophysiology. The first patient had an intracerebral hemorrhage as a result of thrombocytopenia, disseminated intravascular coagulation, and platelet dysfunction. The second case was a case of critical illness polyneuropathy complicating a long ICU stay. In these cases, the neurological complications were secondary to systemic complications and long ICU stay. Autopsy studies are needed to further understand the pathological mechanism.

## 1. Introduction

Middle East Respiratory Syndrome Coronavirus (MERS-CoV) was first identified and isolated in Jeddah, Saudi Arabia, in a 60-year-old male who presented with acute pneumonia complicated by renal failure and death [1]. Since that report, more than 1600 laboratory-confirmed cases of infection with MERS-CoV have been documented in 26 countries until the end of 2015. Among them, more than 580 died comprising about 35% of the total number of cases [2]. The syndrome generally presents as lower respiratory tract disease that includes fever, cough, and shortness of breath that may progress to acute respiratory distress syndrome (ARDS), multiorgan failure, and death [3]. Neurological complications of MERS-CoV have been reported only once in the literature in three cases from Riyadh, Saudi Arabia [4]. Another article from Saudi Arabia reported confusion at presentation among the symptoms of 18 (25.7%) out of 70 confirmed MERS-CoV cases [5]. In this paper, we report two cases of neurological complications of MERS-CoV that affected both the central and peripheral nervous system and we hypothesize the pathophysiology.

## 2. Method

We retrospectively reviewed all MERS-CoV cases admitted at King Abdulaziz Medical City, Jeddah, since the onset of the epidemic in 2012. We identified a total of 120 confirmed cases of MERS-CoV infection. Two patients with neurological complications of MERS-CoV were the subjects of our study. They were admitted to different wards and were managed by different medical teams prior to admission to the intensive care unit (ICU). The clinical, laboratory, and radiological findings of these cases were reviewed. Testing for MERS-CoV was performed using real-time reverse transcription polymerase chain reaction (RT-PCR). This study was approved by the institutional review board (IRB) of King Abdullah International Medical Research Center (KAIMRC), and since this is an observational study, the consent was waived as per the institutional policy.

## 3. Patient 1

**Figure 1:**
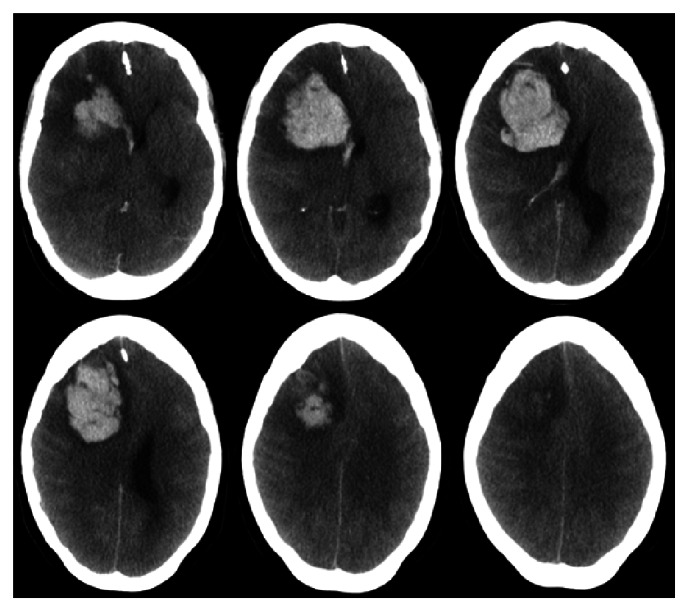
CT of the brain showing right frontal lobe intracerebral hemorrhage with massive brain edema, midline shift, and intraventricular extension.

A thirty-four-year-old female, who was newly diagnosed with diabetes mellitus, presented to the emergency room with a history of high-grade fever of one-day duration. Fever was documented at home and relieved by oral paracetamol. She denied any history of cough or shortness of breath but complained of generalized bone pain and fatigue. Systemic examination showed a febrile ill-looking lady with no lymph node enlargement or skin rash. Chest examination showed decreased air entry bilaterally with crepitation. Neurological examination was normal including higher mental functions, cranial nerves, and motor system, sensory system, and coordination. Laboratory investigations on admission revealed white blood cells of 4.7 with lymphopenia, hemoglobin of 11.3, platelets 203, ESR 47, and CRP 56.5. Chest imaging showed right lung homogenous opacity and the patient was started on intravenous hydration, tazocin, and azithromycin. RT-PCR came back positive for MERS-CoV from sputum. She started to improve, and her condition was stabilized. Unfortunately, two weeks following admission, the patient developed a severe headache, nausea, and vomiting. Few hours later, her consciousness level deteriorated and GCS dropped to 3/15. Urgent CT showed right frontal lobe intracerebral hemorrhage with massive brain edema and midline shift (Figure 1). She was intubated and mechanically ventilated, and she received intravenous mannitol and dexamethasone. Laboratory investigations revealed pictures of disseminated intravascular coagulation including thrombocytopenia and prolonged coagulation profile. Unfortunately, she started to develop multiorgan failure and signs of irreversible brain stem dysfunction and she died two months later.

## 4. Patient 2

A twenty-eight-year-old male, an orthopedic resident, presented to the emergency room with four-day history of fever, generalized myalgia, dizziness, and productive cough. He gave history of contact with a confirmed case of MERS-CoV. He was admitted to an isolated room as a case of acute viral illness with bronchitis and was started on supportive and symptomatic treatment, azithromycin and oseltamivir. He was fully investigated and on day 13 after admission, RT-PCR for MERS-CoV was positive from respiratory secretions. Since admission, his fever never remitted and respiratory functions were progressively deteriorating with desaturation and requirement of high oxygen supplement. On the 4th day after admission, he was intubated and mechanically ventilated and transferred to the ICU. The patient had a stormy course in the ICU, with secondary bacterial pneumonia for which antibiotics were given in full courses. He eventually improved, extubated, and transferred to the floor. In the floor, neurology consultation was requested because of weakness in both legs and inability to walk with numbness and tingling in stocking distribution. He was investigated throughout including neuroimaging, CSF analysis, nerve conduction velocity (NCV) studies, and EMG. MRI of the whole spine and CSF analysis were normal. NCV studies showed low amplitude with normal latency and conduction velocity especially in the lower extremity, which indicated length dependent axonal polyneuropathy. The final diagnosis was critical illness polyneuropathy complicating a long ICU stay. He received intravenous immunoglobulins 400 mg/kg daily for five days and had his respiratory functions closely monitored. He also received extensive daily physiotherapy and was sent home 40 days after admission. He was seen six months later in the clinic, and he was slowly improving.

## 5. Discussion

Coronaviruses are a family of enveloped, single-stranded, positive-sense RNA viruses that are prevalent in bats and can affect many other species including humans. The name* corona* denotes the crown-like appearance of the surface projections of the virus under the electron microscope. They may cause respiratory, gastrointestinal, hepatic, and neurological diseases in various species [1]. They are grouped into four different genera which are alpha, beta, gamma, and delta coronaviruses. There are six types of coronaviruses that afflict humans and thus are called human coronaviruses (HCoV) which are HCoV-229E, HCoV-OC43, HCoV-NL63, HCoV-HKU1, SARS-CoV, and MERS-CoV (Table 1) [6]. Bats are thought to be the natural reservoir of coronaviruses, and the viruses can spread to human through an intermediate reservoir. These viruses have been proven to have the ability to cross the species barrier to infect humans and other animals [7]. The human infection by coronaviruses has been mostly mild and harmless except in SARS-CoV and MERS-CoV where they cause severe morbidity and mortality. Among the human coronaviruses, HCoV-229E, HCoV-OC43, SARS-CoV, and more recently MERS-CoV were proven to be associated with neurological diseases [4, 5, 8].

MERS-CoV first appeared in September 2012 in Saudi Arabia in a 60-year-old male who died from respiratory and renal failure and multiorgan damage [1]. It is thought to have originated from bats and transferred to humans through dromedary camels as an intermediate host. Dromedary camels are part of the culture and economic resources for many businessmen and low-income citizens in Saudi Arabia. Although bats are found in large number in Africa, America, Asia, and Europe, they have been also found in small numbers scattered in caves and mountains in Saudi Arabia [9]. The disease in camels can be just as the common cold. However, it may lead to catastrophic effects in humans. In humans, the virus causes a lower respiratory tract disease and may progress to ARDS, multiorgan failure, and death in severe cases. The elderly and patients with multiple comorbidities appear to be more vulnerable and carry a bad prognosis [3]. The most affected country was Saudi Arabia with more than three-quarters of the confirmed cases in the world. There have been 1280 laboratory-confirmed cases in Saudi Arabia until the end of 2015 [10]. The mode of transmission is not understood thoroughly, but it is thought to transmit by lengthy close contact with an infected human or camel. The mean incubation period of MERS-CoV is five to six days, and it ranges from two to sixteen days [3]. To date, there is no effective treatment for MERS-CoV, and the cases are treated supportively depending on the patient's need [11]. There have been some endeavors to develop a vaccine, but to date there in no effective and approved vaccine for MERS-CoV [12].

Viruses, in general, may enter the brain and spinal cord through either hematogenous spread or retrograde neuronal dissemination. The hematogenous spread occurs through viremia (the presence and multiplication of a given virus in the blood stream). On the other hand, retrograde viral spread occurs when a given virus infects neuronal tissue in the periphery with subsequent spread to the CNS using transport mechanisms within the neurons to gain access to the affected vulnerable areas. Examples of the latter type include rabies and herpes simplex virus encephalitis [13]. Although studies have shown that some coronaviruses possess neurotropic and neuroinvasive properties in various hosts including humans, pigs, and rodents, MERS-CoV has never been isolated from neural tissues or fluids in affected human beings [8]. This could be due to the resistance of coronaviruses to culture by in vitro culture systems. In addition, routine viral culture services for coronaviruses is not available in most clinical laboratories and the process of isolation requires the use of labor-intensive embryonic organ cultures, which is time-consuming [14]. Some reports explained the mechanism by which coronaviruses reach the nervous system which is mainly through the hematogenous route where the virus can either remain dormant for a period before it can infect the endothelial cells of the blood-brain barrier or infect white blood cells that will become the reservoir for the dissemination to other sites [8]. This has never been proved by research. In addition, the blood-brain barrier has several protective characteristics to prevent viruses from entering the brain. In a recent communication by Joob and Wiwanitkit, they proposed that the size of MERS-CoV (150–320 nm) is an obstacle to enter through the 1 nm pore size within the blood-brain barrier [15]. From our point of view and through reviewing the neuroimaging studies, we did not find any meningeal enhancement in most of the cases which supports this theory. We are proposing a different theory which is the autoimmune theory with several involvements of the neural tissues and blood vessels through autoreactive T-cells recognizing both viral and myelin antigens as similar molecules. This immune response that participates in induction or exacerbation of neuropathologies occurs specifically in genetically susceptible individuals [16]. This theory is predominant in explaining the neurological complications of other viruses such as the pandemic influenza A H1N1 pdm09 [17]. This has therapeutic implications which include the possible improvement with the early use of pulse steroid therapy and intravenous immunoglobulin before tissue damage occurs. Steroids and immunoglobulins have been reported previously in the literature in the treatment of SARS-CoV infected patients along with ribavirin. They were not proven to be effective or reduce the morbidity or mortality of the disease. In fact, four studies reported harm due to steroid therapy in terms of avascular necrosis and steroid-induced psychosis in SARS-CoV infected patients [18]. Steroids were also used empirically for the treatment of some MERS-CoV infected patients without proven benefit or positive effect observed [19]. However, we believe that using steroids in MERS-CoV infected cases that present with or develop neurological complications will be beneficial in reducing the mortality and helping with the disease course due to their known benefits in the treatment of neurological diseases. The lack of autopsy studies published in the literature on this topic may participate in keeping the explanation of neurological complications of coronaviruses unclear.

In our cases, none of the theories mentioned above are contributory since our patients presented with sequelae of systemic complications. The first case was explained by an intracerebral hemorrhage as a result of thrombocytopenia, disseminated intravascular coagulation, and platelet dysfunction. The second case was a case of critical illness polyneuropathy complicating a long ICU stay. He made a full recovery with supportive measures, immune modulating treatment, and physiotherapy. The three cases reported in the literature previously along with our cases are summarized in Table 2.

## 6. Conclusion

MERS-CoV infection is a serious disease that affects multiple organs and causes pulmonary, renal, hematological, and gastrointestinal complications. MERS-CoV has created an alarming anxiety in the health sector due to the number of confirmed cases and deaths, especially that there is no treatment available to counterattack this viral infection. Health authorities have released various guidelines to prevent transmission of the disease. Research and studies are ongoing to find the cure or, perhaps, ways to properly manage the case and minimize morbidity and mortality incidence of the disease. There have been some suggested mechanisms through which this virus affects the central nervous system, but the exact mechanism is not thoroughly understood. Autopsy studies are needed to further understand the mechanism.

## Figures and Tables

**Table 1:** The human corona viruses (HCoV).

Virus	Year of discovery	Reservoir	Receptor	Cell type infected	Mode of transmission	Clinical presentation	Disease type	Severity and prognosis
OC43	1966	Bats	Receptor unknown, sialic acid and HLA class 1 involvement	Ciliated airway epithelial cells, macrophages in culture, and neuronal cells	Droplets	Coryza, cough, and fever	Upper respiratory infection, gastrointestinal infection, and pneumonia	Mild and harmless

229E	1967	Bats and camelids	APN	Nonciliated airway epithelial cells, human monocytes, and neuronal cells	Droplets	Coryza, cough, and fever	Upper respiratory infection, gastrointestinal infection, and pneumonia	Mild and harmless

NL63	2004	Bats	ACE2	Ciliated airway epithelial cells	Droplets	Fever, cough, sore throat, and rhinitis	Upper and lower respiratory infection, associated with croup in children	Mild and harmless

HKU1	2005	Bats	Unknown	Ciliated airway epithelial cells	Droplets	Rhinorrhea, fever, coughing, wheezing, and myalgia	Upper respiratory infection and pneumonia, enteric symptoms	Mild and harmless

SARS	2003	Bats, raccoon dogs, and civet cats	ACE2, role for DC-SIGN also known as CS209	Epithelial cells, ciliated cells, and type II pneumocytes	Droplets	Malaise, headache, chills, myalgia, fever, cough, and dyspnea	Lower respiratory infection, pneumonia, diffuse alveolar damage, and ARDS	Severe respiratory illness with high morbidity and mortality

MERS	2012	Bats and dromedary camels	DPP4 also known as CD26	Airway epithelial cells, renal epithelial cells, and dendritic cell	Droplets	Fever, cough, and breathing difficulties	Lower respiratory infection, pneumonia, ARDS, renal failure, and multiorgan failure	Severe respiratory illness with high morbidity and mortality

HLA: human leukocyte antigen, APN: aminopeptidase N, ACE2: angiotensin converting enzyme 2, DC-SIGN: dendritic cell-specific intercellular adhesion molecule-3-grabbing nonintegrin, CD209: cluster of differentiation 209, DPP4: dipeptidyl peptidase 4, CD26: cluster of differentiation 26, ARDS: acute respiratory distress syndrome.

**Table 2:** Patients with neurological complications of Middle East Respiratory Syndrome Coronavirus (MERS-CoV).

Patient	Age/sex	Presenting symptoms	Comorbidities	Diagnosis	Treatments	Outcome	Cause of death
Patient 1 [4]	74/M	Ataxia, vomiting, confusion, and fever	Diabetes, hypertension, and dyslipidemia	Acute disseminated encephalomyelitis (ADEM)	Broad-spectrum antibiotics, oseltamivir, bronchodilators, methylprednisolone, intravenous sedation, neuromuscular blockers, inhaled nitric oxide, vasopressors, renal replacement therapy, peginterferon alpha-2b and ribavirin	Death	Deep coma, poor overall condition, and worsening cardiovascular and respiratory status

Patient 2 [4]	57/M	Flu-like illness, fever, and a gangrenous toe	Diabetes, hypertension, and peripheral vascular disease	Bilateral anterior cerebral artery stroke	Broad-spectrum antibiotics	Death	Severe shock, acute kidney injury, and multiple cardiac arrests

Patient 3 [4]	45/M	Productive cough, dyspnea, rigors, fever, and diarrhea	Diabetes, hypertension, chronic kidney disease, and ischemic heart disease	Encephalitis	Broad-spectrum antibiotics, oseltamivir, renal replacement therapy, neuromuscular blockers, nitric oxide, vasopressors, peginterferon alpha-2b and ribavirin	Recovery	Not applicable

Patient 4	34/F	Fever, generalized bone pain, and fatigue	Diabetes mellitus	Intracerebral hemorrhage	Intravenous hydration, tazocin, azithromycin, mannitol, and dexamethasone	Death	Multiorgan failure and intracerebral hemorrhage with massive brain edema

Patient 5	28/M	Fever, generalized myalgia, dizziness, and productive cough	None	Critical illness polyneuropathy	Azithromycin, oseltamivir, antibiotics, intravenous immunoglobulins, and physiotherapy	Recovery	Not applicable
